# Direct microfabrication of oxide patterns by local electrodeposition of precisely positioned electrolyte: the case of Cu_2_O

**DOI:** 10.1038/srep27423

**Published:** 2016-06-03

**Authors:** P. Wang, R. C. Roberts, A. H. W. Ngan

**Affiliations:** 1 Department of Mechanical Engineering, The University of Hong Kong, Pokfulam Road, Hong Kong; 2 Department of Electrical and Electronic Engineering, The University of Hong Kong, Pokfulam Road, Hong Kong

## Abstract

An efficient technique for writing 2D oxide patterns on conductive substrates is proposed and demonstrated in this paper. The technique concerns a novel concept for selective electrodeposition, in which a minimum quantity of liquid electrolyte, through an extrusion nozzle, is delivered and manipulated into the desired shape on the substrate, meanwhile being electrodeposited into the product by an applied voltage across the nozzle and substrate. Patterns of primarily Cu_2_O with 80~90% molar fraction are successfully fabricated on stainless steel substrates using this method. A key factor that allows the solid product to be primarily oxide Cu_2_O instead of metal Cu – the product predicted by the equilibrium Pourbaix diagram given the unusually large absolute deposition voltage used in this method, is the non-equilibrium condition involved in the process due to the short deposition time. Other factors including the motion of the extrusion nozzle relative to the substrate and the surface profile of the substrate that influence the electrodeposition performance are also discussed.

Developing cost-effective capabilities to fabricate materials into desired geometries has always been a prime focus of materials research. For metals and ceramics, macro-scale parts are formed using thermal and mechanical methods such as casting, powder-metallurgy sintering, and conventional subtractive machining^1^,^2^. Micro-sized structures, such as those used in thin-film electronics or small devices, are routinely made using techniques including physical and chemical vapor deposition, combined with photolithographic patterning^3^,^4^,^5^,^6^. However, for the “meso-scale” in the sub-millimeter range, these methods are not cost effective^7^. At this length scale, micro-casting or sintering would be very expensive due to the costly mold-making process, and machining may require the use of laser technology which again is costly. Conventional lithography methods also become practically difficult if the substrate dimensions well exceed the micro-scale; for instance making one-dimensional structures with lengths well beyond the micron regime would be challenging and expensive. Additionally, subtractive manufacturing like machining, and multi-step methods such as casting or lithography, produce significant amount of material or reagent wastage which is detrimental to not only the processing cost but also the environment.

In this paper, we propose and demonstrate a novel technique for fabricating 2D ceramic patterns, using selective electrodeposition with a controlled volume of liquid electrolyte of nearly the same shape as the final solid product. Essentially, in this method, a micro volume of liquid electrolyte is delivered onto a conductive substrate by a conductive syringe needle. Meanwhile, the electrolyte volume, triggered by an applied voltage between the substrate and the syringe needle that also serves as the anode, is converted instantly into ceramic by an electrochemical reaction. By continuously delivering the electrolyte and moving the substrate relative to the electrolyte delivering needle, the continuous electrodeposition process allows any solid pattern to be made onto the substrate. When combined with digital manufacturing, the technique can, in a single step, provide an efficient process for fabricating 2D ceramic patterns onto conductive substrates for a wide range of applications.

For ceramic pattern production, this technique is more viable than other contemporary fabrication methods such as selective-laser sintering (SLS)^8^ or inkjet printing^9^,^10^,^11^. Due to their high melting temperatures, most ceramics cannot be directly sintered by the SLS process, while indirect approaches involving first coating the ceramic powder with a temporary binder followed by post-SLS thermal treatments^12^ are complicated and not cost effective. Likewise, inkjet printing only produces a temporary ink pattern of a sub-micron ceramic particle suspension or metallic precursor, and the printed ink also needs to be further treated to produce the final product^9^. Thus, the method still requires rather complex procedures, especially for preparing the suspension or precursor, and fabricating the sub-micron particles can be costly. Our new technique is also distinctively different from other selective electrodeposition methods in which the workpiece is submerged into a bulk bath of electrolyte and either a mask is applied^3^,^4^,^5^,^6^ or some sacrificial layer is created^13^ onto the workpiece to shield off the regions that need not be electrodeposited. Such masked electrodeposition methods all involve multiple steps including the formation and removal of the patterned resist before and after the actual electrodeposition which actually represent a major part of the production cost^13^. More importantly, the wider use of these methods is limited by the fact that the dimensions of patterns that can be deposited are constrained by the size of the electrolyte bath as well as that of the patterned mask that can be made, i.e. such selective electrodeposition methods would not be applicable to making long wires or lines. For the latter, Hu and Yu have demonstrated the production of metallic micro-wires by manipulating the meniscus of a micro-stream of electrolyte in 3D^14^, but since the electrochemical reaction in this case is sustained by the conductivity of the already formed wire length connected to the meniscus, this method would not be applicable to ceramics with poorer conductivity. Rajput *et al*. also studied a mask-less electrodeposition technique by impinging an electrolyte jet against the substrate surface^15^. However, since the electrolyte is made to splash onto the workpiece surface, the boundary between the electrodeposited and un-electrodeposited region tends to be irregular, which limits the resolution of the pattern to be formed. On the contrary, the main concept advocated in our new approach here is that ceramic patterns can be electrodeposited in one single step from the least amount of liquid electrolyte manipulated into the final shape – in other words, such a concept stipulates the least wastage of reagents among all possible electrodeposition methods.

To demonstrate the feasibility of our method, patterned deposition of cuprous oxide (Cu_2_O) is reported in this paper. Cu_2_O has been well studied for its ability of photovoltaic solar energy conversion^16^,^17^,^18^. In addition, it can also be used as photo-catalyst for converting water into hydrogen and oxygen under illumination of visible light^19^, as catalyst for conversion of organic pollutants such as ethanol^20^, and as protective coatings for the graphite electrode in PC-based lithium ion batteries^21^. In this study, patterns of Cu_2_O are electrodeposited on stainless steel substrates. For electrodeposition using electrolyte bath, factors such as cathodic polarization, current density and *pH* value of the electrolyte are known to influence the product obtained^16^,^18^,^22^,^23^,^24^. However, our method involves much smaller volumes of electrolyte and fast motion of the anode, and such factors were found to result in non-equilibrium condition which is actually a key factor for the production of primarily oxide in the electrodeposition product.

## Experimental Setup

**Figure 1:**
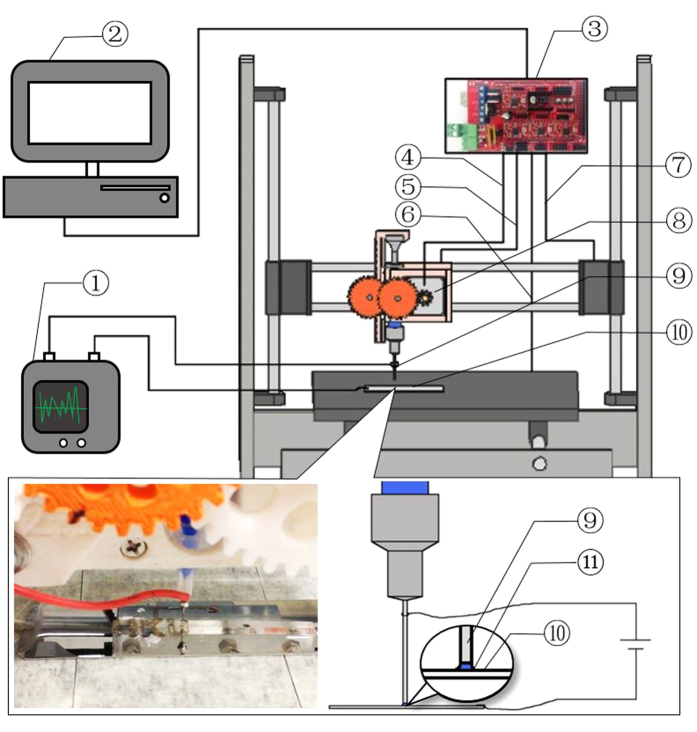
Schematic of the setup, numbered components being: ➀ Electrochemistry workstation, ➁ Computer, ➂ Motion controller, ➃ Extrusion Control, ➄ X-direction motion control, ➅ Y-direction motion control, ➆ Z-direction motion control, ➇ Extruder, ➈ Syringe needle (anode), ➉ Substrate (cathode), ⑪ Electrolyte. Inset shows the letters “HKU” being made onto the stainless steel substrate (a video showing the process and a photograph of the set-up are available as Supplementary Material).

A computer-controlled system for direct electrodeposition of ceramic patterns was custom designed and built as shown in Fig. 1. The setup comprises an X-Y-Z motion system (Zonestar Prusa i3, Shenzhen Zonestar Innovation Technology Co., Ltd), a modified syringe extruder assembled from consumer-grade 3D-printed components (original design by Shapescribe^TM^, details of modification available in Supplementary Materials Section S1), and an electrochemical workstation (LK2006A, Lanlike).

As shown by Fig. 1, the electrolyte is stored in a syringe inserted into the syringe extruder. To move the syringe needle relative to the substrate, the syringe extruder is installed onto the gantry of the motion system, while the substrate is fixed onto the platform. Then the syringe needle and substrate are connected to the electrochemical workstation with a two-electrode configuration to function as anode and cathode respectively. A voltage is applied from the workstation simultaneously across the needle and substrate when the syringe needle is made to move relative to the substrate and the electrolyte is extruded at certain rate. The voltage activates the extruded electrolyte and deposits a solid material on the substrate exclusively at the area below the syringe needle and covered by electrolyte. The movement of the syringe needle relative to the substrate then leads to selective electrodeposition at controllable locations on the substrate, hence generating a 2D pattern of the deposited material. The applied voltage with respect to (w.r.t.) the anode *E* and velocity of the syringe needle *u* can be varied in order to produce different effects on the electrodeposition process and the product obtained. Further details of the experimental setup are given in the Section S1 of Supplementary Materials.

## Results

**Figure 2:**
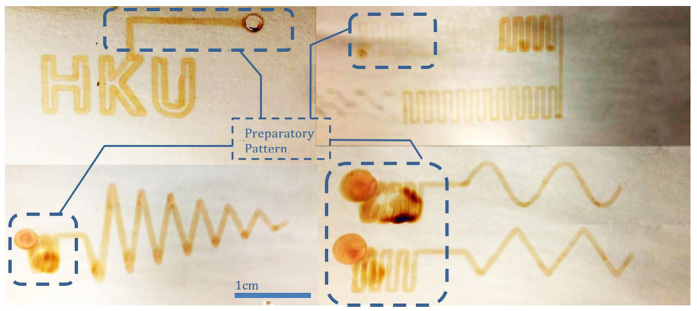
Examples of patterns electrodeposited on stainless steel substrates.

In this work, the deposition of Cu_2_O using an electrolyte containing 0.4 M CuSO_4_ and 3 M sodium lactate^18^ on stainless steel substrates was studied using the setup described above. Details of the experimental and analysis procedures are given at the end of this paper. The inset of Fig. 1 shows a magnified snapshot of the syringe needle during the process of writing a pattern (a video showing the process is available as Supplementary Material). It is remarkable to note that while the Cu^2+^ based electrolyte in the syringe was blue in color, the deposited product on the stainless steel substrate instantly developed a brownish color, indicating the occurrence of an electrochemical reaction. Examples of patterns successfully fabricated with the present electrodeposition method are shown in Fig. 2. To ensure that the electrodeposition process was conducted under stable conditions, typically, a preparatory pattern, as indicated by circled regions in Fig. 2, was made to allow the extrusion and electrodeposition systems to stabilize before depositing the desired pattern. Among all the conditions studied, electrodeposition with speed of nozzle relative to the substrate *u* = 200 mm/min and applied voltage w.r.t. the cathode *E* = −2.5 V was found to obtain the best result. In this report, a negative sign for *E* indicates that the substrate serves as the cathode, namely, the potential applied there is negative relative to the nozzle, while a positive sign indicates the opposite. With the size of the syringe needle and the deposition conditions described above, the width *w* of the deposited lines was measured as ∼600 μm. As examples, patterns resembling a sine wave, triangular wave, and letters “HKU” were fabricated with the optimal combination. Cyclic patterns were also made, in which different segments were deposited at different combinations of *E* and *u*, thus resulting in different color darkness.

**Figure 3:**
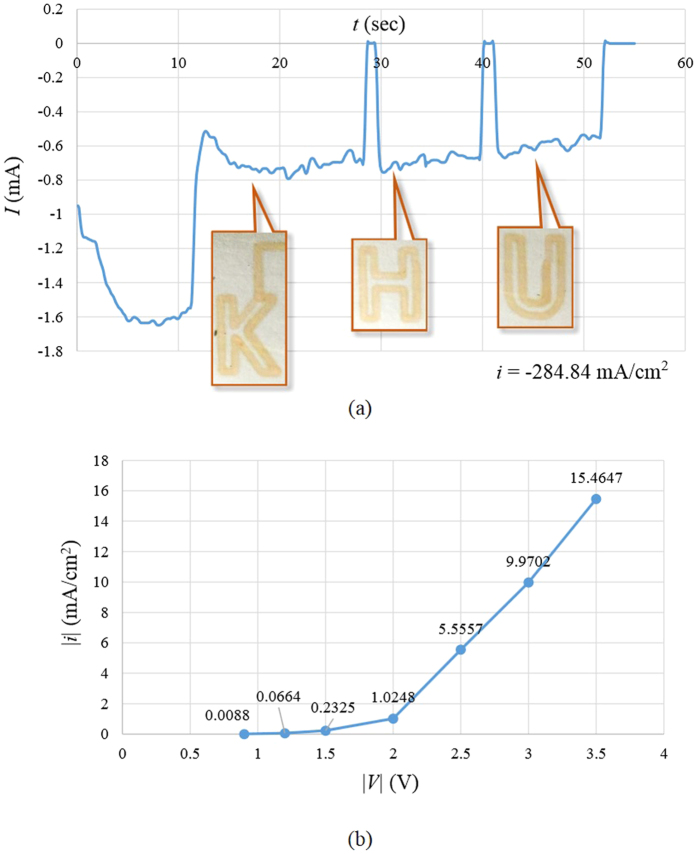
(**a**) Current vs time during the electrodeposition of the “HKU” pattern. (**b**) Current density vs voltage (both in magnitudes) at writing speed *u* = 100 mm/min.

**Figure 4:**
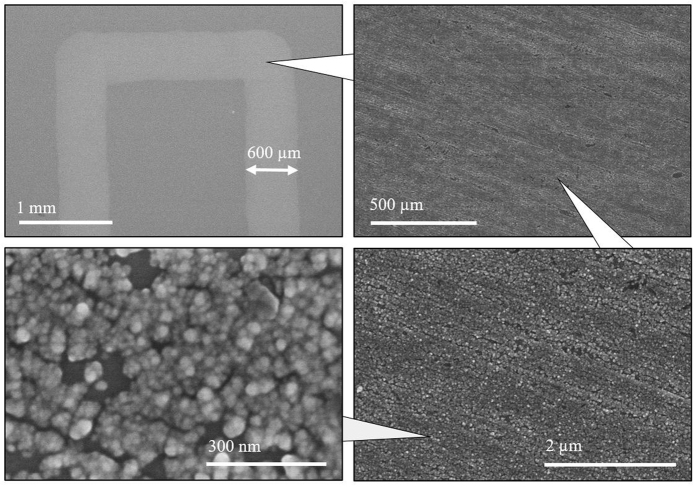
SEM micrographs of a typical fabricated pattern at different magnifications.

The current profile recorded during the electrodeposition of the “HKU” pattern is shown in Fig. 3(a). At the beginning, a previously extruded droplet of electrolyte, as part of the preparatory pattern, was first electrodeposited, which corresponds to the recorded current *I* at ~−1.4 mA. As the nozzle started to move relative to the substrate, a decrease in the magnitude of the recorded current was observed, since the area of electrodeposition decreased from the initial contact area between droplet and substrate, to the area directly below the anode. During the electrodeposition process, *I* became stabilized between the value of −0.6 A and −0.8 A, with some fluctuations possibly caused by the vibration of the motion system. Between the processes of printing different letters, the nozzle was first lifted from the end of the previous letter, translated and then lowered to the beginning of the next letter. During the lifting and translating process, there was no electrolyte between the nozzle and substrate, which resulted in open circuit, hence a recorded current of around zero. At *u* = 100 mm/min, the relation between current density *i* and deposition voltage *E*, both in absolute values, is shown in Fig. 3(b). Figure 4 shows the SEM images of the fabricated pattern. It can be seen that the deposited product forms a thin film of nano-particles clustered together. Clear contrast exists between the electrodeposited and un-electrodeposited regions. The particles exhibited irregular shapes and different sizes in the range of 10~100 nm. It was observed that several locations were not coated with the deposition product, as shown in the image at the highest magnification in Fig. 4. The coating performance was measured by the area fraction *μ* of regions covered by product, namely,


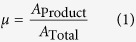


*μ* was found to be influenced by *E* and *u*, as shown by Fig. S2 in Supplementary Materials.

**Figure 5:**
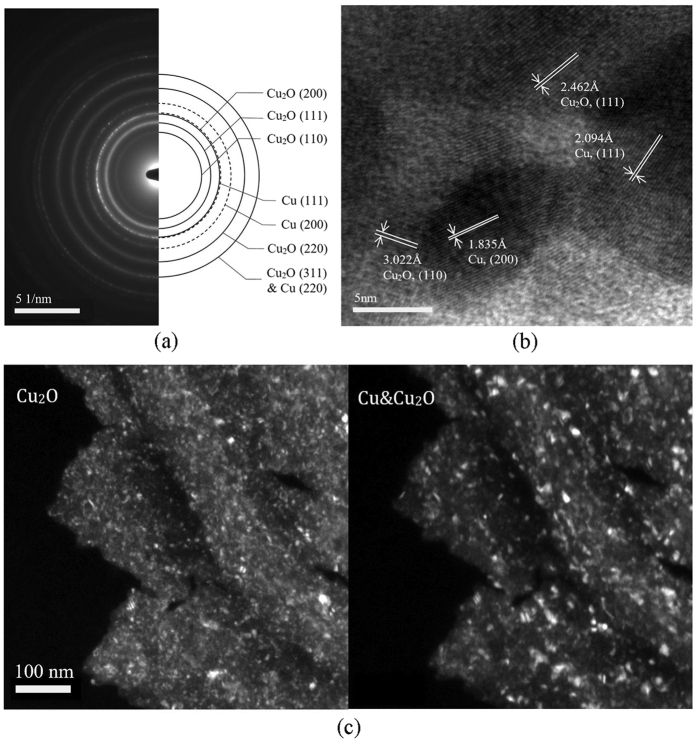
Selected area electron diffraction pattern (**a**), high-resolution TEM (**b**) and dark-field images (**c**) of product electrodeposited with *E* = −2.5 V and *u* = 200 mm/min.

Electron diffraction performed on the sample that was electrodeposited with *E* = −2.5 V and *u* = 200 mm/min revealed primarily Cu_2_O (80~90% molar fraction, see Section S3 of Supplementary Materials) with the coexistence of Cu. The diffraction pattern, shown in Fig. 5(a), consists of continuous and dotty rings, which indicate the existence of tiny and relatively larger crystals respectively. By comparing the measured atomic plane spacing and intensity of each ring with theoretical data (JCPDS, No. 85–1326 & No. 78–2076)^25^, it was found that the continuous rings correspond to Cu_2_O, while the dotty rings correspond to Cu, as indicated in Fig. 5(a). The result was further confirmed by dark-field transmission electron microscope (TEM) imaging, as shown in Fig. 5(c). The image produced jointly from reflections Cu(111), Cu(200), and Cu_2_O(200) shows predominantly large crystal particles (right panel of Fig. 5(c)), while that from reflections Cu_2_O(110) and Cu_2_O(111) shows tiny crystals (left panel of Fig. 5(c)). The co-existence can also be seen from high-resolution TEM imaging (Fig. 5(b)) which shows a few sets of lattice fringes. With fast Fourier transform (FFT) analysis, the values of atomic plane spacing measured from the lattice fringes were found to agree with theoretical values for both Cu and Cu_2_O^25^ as indicated in Fig. 5(b). In addition to electron diffraction, grazing incidence X-ray diffraction (XRD) further confirmed that the deposited product contains Cu_2_O, as shown in Fig. S3 in Supplementary Materials.

Energy-dispersive X-ray spectroscopy (EDX) performed on the syringe needles inside the field emission scanning electron microscope (FE-SEM) revealed that there was no significant change in their chemical composition after being used as the anode. Table S2 in the Supplementary Materials compares the chemical compositions by weight percentage (wt%) of the used and new syringe needles. From the comparison, it can be concluded that insignificant amounts of metallic elements Cr and Ni within the syringe needle were released into the electrolyte during the electrodeposition process, and so the product should not be contaminated. EDX analysis of the product shows no traces of Cr, although Ni was detected due to the Ni mesh used, as shown in Table S3 in the Supplementary Materials.

## Discussion

During the electrodeposition process, the possible reactions that may occur at the cathode are:


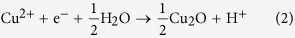



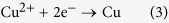



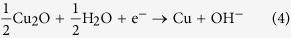


To determine the reactions that actually occur at the cathode, the equilibrium potentials of the three reactions are calculated as follows. Reactions with equilibrium potential higher than the applied cathodic potential are thermodynamically favorable. According to Nernst equation, for reaction (2),


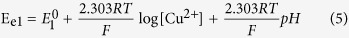


Similarly, for reactions (3) and (4),


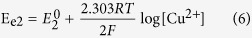



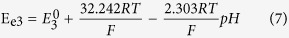


Here 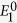, 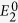 and 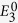 are standard electrode potentials corresponding to each reaction, *R* is universal gas constant, *T* is temperature, *F* is Faraday’s constant, and [Cu^2+^] represents the concentration of Cu^2+^. The values for 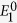, 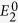 and 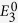 are 0.203 V, 0.340 V and −0.360 V w.r.t. standard hydrogen electrode (SHE) respectively^26^. Due to the short duration of the electrodeposition process, the *pH* value of the electrolyte is assumed to be constantly 12 for simplicity of the calculation. Since Cu^2+^ form complexes with lactate ions, the concentration of Cu^2+^ cannot be directly calculated. Instead, a possible range of concentration (10^−5^~1 mol/L) is used to determine the span of equilibrium potentials for the three reactions, all of which are found to be larger than −0.48 V w.r.t. SHE. With applied voltage *E* = −2.5 V, which is approximately −1.7 V w.r.t. SHE and much larger, in terms of absolute value, than the calculated equilibrium potentials, all the three reactions can occur at the cathode.

Given sufficient time, all the Cu_2_O produced by reaction (2) should eventually be consumed and turned into Cu. Indeed, according to the Pourbaix diagram of Cu system, at applied voltage of −1.7 V w.r.t. SHE, the obtained product should be purely Cu^27^. Experimentally, co-deposition of Cu_2_O and Cu was observed in previous studies with a similar electrolyte as the present work, except that a much lower voltage, in terms of absolute value, was used at the cathode^22^,^28^. In the present work, however, the results in Fig. 5 clearly indicate the co-existence of Cu along with Cu_2_O in the deposited product. The discrepancy between the previous and current work is likely a result of the short duration of electrodeposition in this study. In our experiments, the electrodeposition performed at *u* = 200 mm/min at a location with size of 1 mm × 0.6 mm (length × width) lasted for less than 0.5 sec, which is much shorter than the durations in previous studies. Such a short duration will likely prevent the system from reaching equilibrium and obtaining the equilibrium product of Cu according to the Pourbaix diagram. More specifically, before the produced Cu_2_O at a given location is converted into Cu via reaction (4), the anode has already moved to another location, stopping the process from reaching equilibrium at the current location. This explains why the deposition product is composed primarily of Cu_2_O with a small amount of Cu, which might actually help improve the functional performance of the deposited product. In fact, it has been reported that, by mixing with Cu, the performance of the photovoltaic and photo-catalytic functions of Cu_2_O can be significantly improved^28^,^29^,^30^,^31^,^32^. To verify the proposed hypothesis, electrodeposition without the motion of the needle relative to the substrate with *E* = −2.5 V and duration of 0.5 min was also conducted. The product exhibited a significantly larger amount of Cu, with molar fraction of Cu_2_O decreased to 42.34%.

It was found that the areal coverage *μ* increases with more negative *E*, and decreases with larger *u*, as shown in Fig. S2 in Supplementary Materials. According to Faraday’s law of electrolysis, the product deposition rate is proportional to the absolute value of the current density |*i*|, i.e.


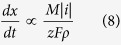


where *x*, *M* and *ρ* are respectively the thickness, molecular mass and density of the deposited product, and *z* is the number of moles of electrons involved in forming one mole of the product. Equation (8) suggests that with increased current density, the rate of electrodeposition is also elevated. Meanwhile, the Tafel equation describes the influence on current density *i* from the overpotential *η*– the difference between the equilibrium and applied potential, i.e.


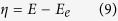



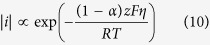


where *α* is the fraction of energy from the overpotential that assists cathodic reaction. By lowering the applied potential *E* to a more negative value, *η* becomes more negative, so that 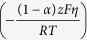 and hence the absolute value of the current density increase. This theoretical relation corresponds well with the experimental observation in Fig. 3(b). Therefore, with the applied voltage *E* being more negative, the amount of product made per unit time increases, which results in a higher portion *μ* of cathode being coated by the product.

The effect from nozzle motion speed *u* on *μ* is likely due to the reduced electrodeposition time. By integrating *x* w.r.t. *t* in Eqn. (8), it can be seen that the amount of product being deposited is proportional to the duration of electrodeposition. At higher *u*, the duration of electrodeposition is reduced, thus decreasing the amount of product obtained, i.e. lower value of *μ*.

The deposited product was mainly round crystal with irregular surfaces, as shown in Fig. 4. Such a morphology is rarely seen in previous studies on electrodeposition of Cu_2_O or Cu which commonly reported regular-shaped films or particles with sharp edges and corners^28^,^29^,^33^,^34^. To grow sharp edges, as proposed by Zhao *et al*. a local deficiency of Cu^2+^ supply should be developed^35^. In our experiment, the supply of Cu^2+^ is ample, due to the short duration of the electrodeposition process. Moreover, the motion of the syringe-needle anode constantly stirs the electrolyte, which enhances convection and further supplies the electrode/electrolyte interface with abundant Cu^2+^. Therefore, the local deficiency can hardly be developed, and the grown particles exhibit round shapes without sharp edges or corners.

It was also observed that the size of the crystal particles was insensitive to changes of the applied voltage in the range of *E* ≤ −2.0 V, which can be explained by the well-known kinetic competition between nucleation and growth^36^. For all the different applied voltages, the high overpotential applied during the experiment provided enough driving force for nucleation at almost any site on the substrate. Hence, the number of nucleation sites remains nearly unchanged as voltage varies within the specified range, which implies that the ability of nucleation to compete against crystal growth remains constant as voltage increases. Therefore, the size of the deposited particles remains unchanged even though different voltages are applied. With *E* > −2.0 V, the applied voltage supplied sufficient energy for nucleation, but not for growth, which results in much smaller particles. The change of particle size w.r.t. deposition voltage is shown in Fig. S4 in Supplementary Materials.

In addition to the influence from applied potential and motion of anode, the surface profile of the substrate also affects the structure of the deposited film. It can be seen from Fig. 4 that the pattern of the film coincides with the surface profile of the substrate. Since the surface of the substrate is not perfectly flat, the electric field can be slightly distorted by the protrusions or depressions on the substrate. Therefore, electrodeposition at certain sites is slightly favored over other sites, which would result in the deposition of Cu_2_O in the same pattern of the substrate surface.

As compared to the traditional electrodeposition conducted in bulk electrolytes, in which parameters like ion concentration and *pH* value remain fairly constant, the current setup makes use of a small volume of electrolyte which is also constantly being stirred due to the motion of the anode. Such a configuration difference introduces unconventional changes into the system in terms of agitation of the electrolyte, change in concentration and *pH* value. Due to the limited volume of the electrolyte, a slight amount of Cu^2+^ being consumed will result in a rather significant change in concentration. Likewise, since OH^−^ is also consumed during the reactions, the *pH* value varies as electrodeposition continues. Accompanying the motion of anode is a continuous change of the morphology of the cathode/electrolyte interface as encountered by the anode syringe needle, and the transport mechanism of Cu^2+^, as mentioned above.

These changes result in different behavior of the system from that predicted using theoretical models that have been successfully applied to traditional methods with bulk electrolytes. For instance, as discussed above, the electrodeposited product is different from theoretically predicted results, as the motion of anode prevents the system from reaching reaction equilibrium. Such peculiarity enables the system to achieve unique performance like electrodepositing dense product at high current density during a short period of time.

Finally, it is worthwhile to comment on the preparatory patterns, in the examples such as those shown in Fig. 2. As mentioned above, those preparatory patterns were added to enable the subsequent deposition to achieve stable conditions, but such pre-writing would be undesirable in practical applications. It is believed that the need to carry out significant pre-writing would be greatly reduced if the extrusion system has higher precision control of the extrusion pressure. Future work should therefore be carried out to study this aspect.

## Conclusions

A novel method to electrodeposit metallic and oxide in 2D patterns was developed. This method advocates the concept of “most economical electrodeposition”, by delivering the minimum quantity of liquid electrolyte and manipulating it into the desired shape on the substrate, and turning it into solid deposits by an electro-redox reaction in a single step. Cu_2_O was primarily (80~90% molar fraction) electrodeposited into various patterns on stainless steel substrates by this way. Electron microscopy examination revealed that the deposited pattern was made up of a thin film of densely packed nano-sized Cu_2_O crystals in the range of 10~100 nm with a small amount of Cu. Although the applied voltage exceeded the equilibrium limit for the production of Cu_2_O, the method is still able to produce a majority of Cu_2_O, rather than metallic Cu according to equilibrium, as a result of the fast motion of the anode which prevents equilibrium to be reached. The observed effects from deposition voltage and speed of the anode agree well with considerations from reaction kinetics. The morphology of the product is found to be affected by the motion of the anode and structure of the substrate surface. Since the electrodeposition system introduces factors that cause rather significant changes in parameters like concentration and *pH* values, the electrodeposition performance shows advantageous deviation from traditional systems involving bulk electrolytes, rendering the system the ability to perform selective electrodeposition for novel materials and applications.

## Methods

The syringe used in the present work for delivering electrolyte onto the substrate had a capacity of 3 mL and an inner diameter the needle of 300 μm. The electrolyte used contained 0.4 M CuSO_4_ and 3 M sodium lactate. The pH value was adjusted to 12 using 5 M NaOH solution^18^. By forming complexes with lactate ions, the copper ions are stabilized and do not form precipitates in alkaline environment^21^, which favors producing Cu_2_O at relatively large magnitudes of the deposition voltage. The substrate onto which Cu_2_O was electrodeposited was a stainless steel plate measuring 80 mm × 25 mm × 0.6 mm (length × width × thickness). Before being electrodeposited, the substrate was polished with sandpaper of grit size P4000 and then rinsed in distilled water and ethanol successively. The combinations of applied voltage *E* and velocity of the syringe needle *u* used are shown in Table S1 in the Supplementary Materials. A nominal separation of 150 μm between the nozzle and substrate was maintained during the process.

During the electrodeposition process, the current *I* was measured against time *t* and recorded during the electrodeposition process. The average current density 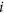 was then calculated as


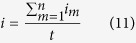


Here, *i*_*m*_ is the current density at a time instant *t*_*m*_ calculated as


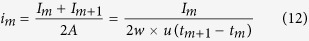


where *I*_*m*_ is the measured current at time instant *t*_*m*_, *A* is the area swept by the extruder during the time period *t*_*m*+1_ − *t*_*m*_, and *w* is the width of the electrodeposited line.

The morphology, internal structure and chemical composition of the product were examined after the selective electrodeposition process. An FE-SEM (Hitachi S-4800) was used to observe (at an acceleration voltage of 5 kV) the morphology of the deposited product. TEM and electron diffraction conducted inside the TEM (Tecnai G2) were employed to examine the microstructure and crystallography of the products respectively. To perform the TEM examination, the deposition product was first carefully desquamated from the substrate using tweezers that had been dipped with ethanol. Then the product was transferred onto a standard TEM Formvar film supported by a nickel mesh. To further confirm the chemical composition of the product, grazing incidence XRD (Rigaku SmartLab) was also performed. To determine the possible reactions that occurred at the anode, the metallic portion of the hypodermic needle was harvested after the experiments and examined with EDX installed on the FESEM to determine the change of the chemical composition within the needle. A new unused syringe needle was also examined to determine the initial chemical composition for comparison.

## Additional Information

**How to cite this article**: Wang, P. *et al*. Direct microfabrication of oxide patterns by local electrodeposition of precisely positioned electrolyte: the case of Cu_2_O. *Sci. Rep.* **6**, 27423; doi: 10.1038/srep27423 (2016).

## Supplementary Material

Supplementary Information

Supplementary Video 1
