# Supplementary material for: Direct microfabrication of oxide patterns by local electrodeposition of precisely positioned electrolyte: the case of Cu2O
Source: Sci Rep. 2016 Jun 3;6:27423. doi: 10.1038/srep27423 (PMC4891777; doi:10.1038/srep27423)
Supplement: Supplementary Information [file srep27423-s1.pdf]

Direct microfabrication of oxide patterns by local electrodeposition of precisely positioned electrolyte: the case of Cu_2O

P. Wang[1], R.C. Roberts[2] and A.H.W. Ngan[1, §]

[1] Department of Mechanical Engineering,
[2] Department of Electrical and Electronic Engineering,
The University of Hong Kong, Pokfulam Road, Hong Kong

[§] Corresponding author (email: hwngan@hku.hk)

Supplementary Materials

S1. Details of experimental setup

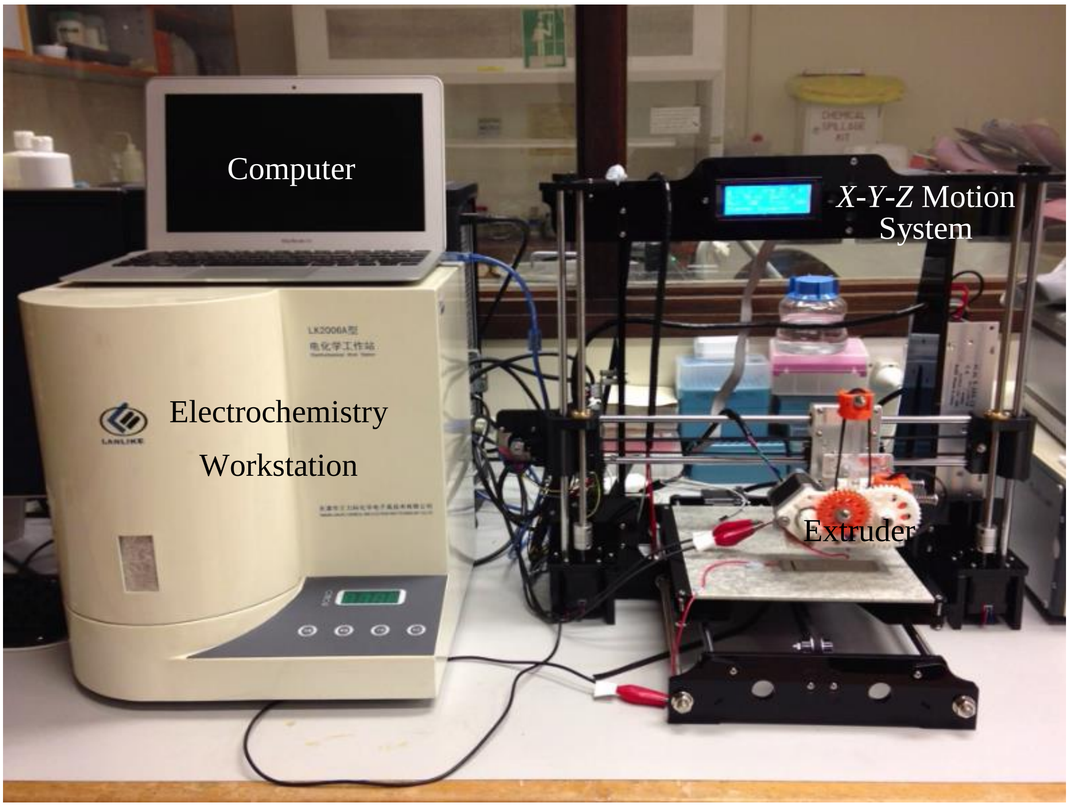

Fig. S1 Experimental setup. The main components of the system are labelled in the picture.

As described in the main text, a computer-controlled system for extruding electrolyte on the substrate was custom built as shown in Fig. S1. In this setup, an X-Y-Z motion system (Zonestar Prusa i3, Shenzhen Zonestar Innovation Technology Co., Ltd) is used to control the positioning of the syringe extruder, from which the electrolyte is delivered onto the desired location of the substrate. The syringe extruder used was assembled from 3D-printed components with original design by Shapescribe^{TM}[1]. The extruder was originally designed for 10 mL syringes, but in the present work a 3 mL syringe was used so as to achieve higher control precision of extrusion. To properly fit the 3 mL syringe into the extruder, a 3D-printed adaptor designed by ourselves[2] was used.

S2. Further experimental data discussed in main text

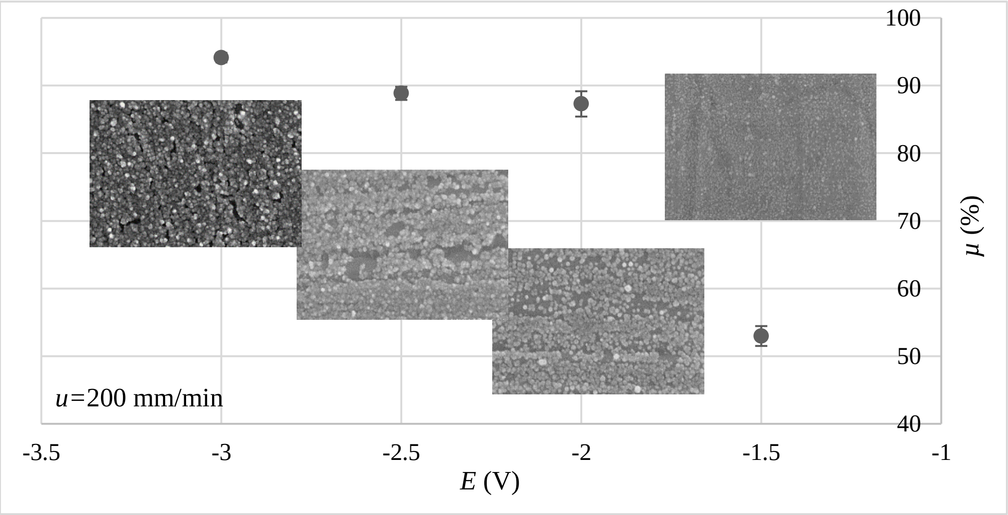

(a)

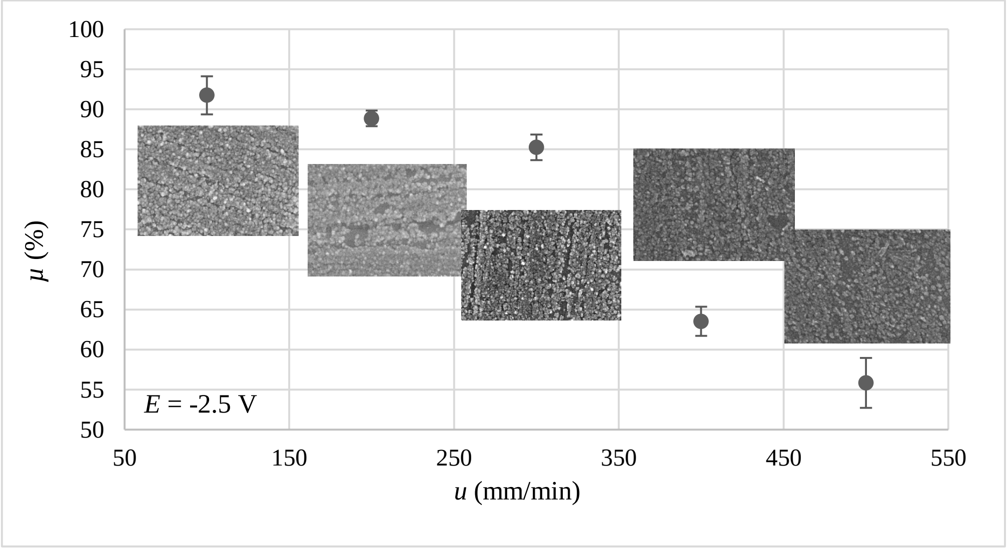

(b)

Fig. S2 – Effects of voltage E and writing speed u on areal coverage of the product. (a) Effect of E at constant u = 200 mm/min. (b) Effect of u at constant E = -2.5V.

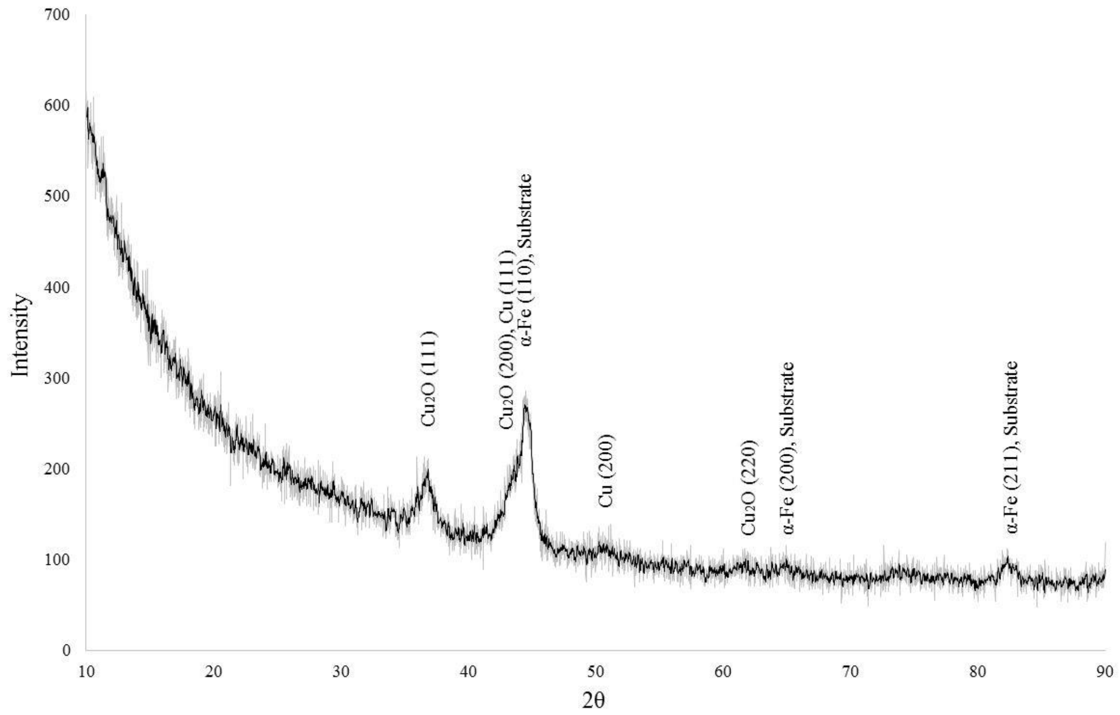

Fig. S3 – Grazing incidence XRD pattern of an area of the product measuring ~ $1\,mm \times 1\,mm$, deposited with E = -2.5 V and u = 200 mm/min. By comparing with standard diffraction pattern of Cu_2O (JCPDS No. 78-2076), Cu (JCPDS No. 85-1326) and α-Fe (JCPDS No. 85-1410)[3], the product consisted of predominately Cu_2O with traces of Cu. The plot in light grey color shows the raw data, while that in black color was obtained by performing averaging smoothing of the raw data to reduce noise.

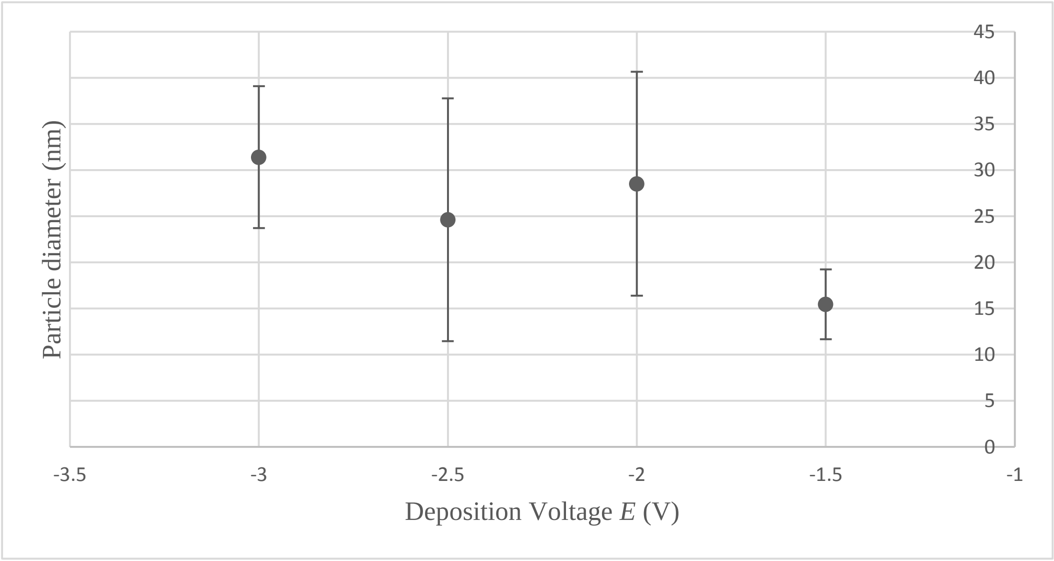

Fig. S4 – Effects of voltage E on particles size. The influence is insignificant with $E < -2.0$ V. With $E > -1.5$ V, particle size decreases with increased voltage.

Table S1 – Combination of Different Voltage and Extruder Velocity

Voltage E (V)	Velocity u (mm/min)
-2.5	100
	200
	300
	400
	500
-1.5	200
-2.0	
-2.5	
-3.0	
-0.9	100
-1.2	
-1.5	
-2.0	
-2.5	
-3.0	
-3.5	

Table S2 – Chemical Compositions of New and Used Syringe Needle

Used			Original		
Element	wt. %	Standard Deviation	Element	wt. %	Standard Deviation
O	6.65	2.21	O	4.18	2.87
Si	0.57	0.10	Si	3.02	0.17
Cr	18.19	0.53	Cr	18.34	0.64
Mn	1.55	0.29	Mn	1.54	0.31
Fe	64.52	1.59	Fe	64.19	1.98
Ni	8.52	0.40	Ni	8.73	0.46

Table S3 – Chemical Compositions of the Product Obtained from EDX Measurement

Element	Weight percentage (wt. %)	wt. % Standard Deviation	Atomic %	Spectrum
O	12.14	0.23	35.44	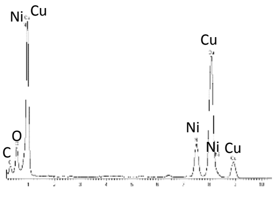
Cu	87.86	0.23	64.56	

Table S4 – Molar fraction of Cu_2O

Sample	$f(Cu_2O)$
E = -2.5 V<br>u = 200 mm/min	79.93%
E = -2.5 V<br>u = 100 mm/min	89.35%
E = -2.5V<br>Stationary	42.34%

S3. Measurement of molar fractions of Cu_2O in the deposited products by electron diffraction

The volumetric ratio of Cu_2O and Cu, $x(Cu_2O)/x(Cu)$, is given by:

$$\frac{x(\mathrm{Cu_2O})}{x(\mathrm{Cu})} = \frac{I_{\mathrm{Cu(200)}}(\mathrm{theoretical})}{I_{\mathrm{Cu_2O(111)}}(\mathrm{theoretical})} \frac{I_{\mathrm{Cu(200)}}(\mathrm{experimental})}{I_{\mathrm{Cu_2O(111)}}(\mathrm{experimental})} \qquad \text{(S1)}$$

where $I_{\mathrm{Cu(200)}}$ and $I_{\mathrm{Cu_2O(111)}}$ are the diffraction intensities of the Cu(200) and Cu_2O(111) reflections, respectively. The theoretical ratio of diffraction intensities in equation (S1) was calculated using the electron diffraction simulation software JEMS[4]. Then, from selected-area diffraction patterns such as that shown in Fig. 5(a) in the main text, the experimental ratio of diffraction intensities in equation (S1) was measured using the image processing software ImageJ[5] after first subtracting the background, which arises from inelastic scattering of electrons, of the diffraction pattern. Then an average intensity value was calculated from the values measured at different locations on the particular diffraction ring. The average intensity value was used to calculate the experimental ratio of diffraction intensities in equation (S1).

From the $x(Cu_2O)/x(Cu)$ ratio calculated from equation (S1), the molar ratio $n(Cu_2O)/n(Cu)$ is obtained as:

$$\frac{n(\mathrm{Cu_2O})}{n(\mathrm{Cu})} = \frac{x(\mathrm{Cu_2O})\rho(\mathrm{Cu_2O})M(\mathrm{Cu_2O})}{x(\mathrm{Cu})\rho(\mathrm{Cu})M(\mathrm{Cu})} \qquad \text{(S2)}$$

where ρ is the density, and M is the molar mass of each phase. The molar fraction of Cu_2O $f(\mathrm{Cu_2O})$ is then given as:

$$f(\mathrm{Cu_2O}) = \frac{n(\mathrm{Cu_2O})}{n(\mathrm{Cu}) + n(\mathrm{Cu_2O})} = \frac{\dfrac{n(\mathrm{Cu_2O})}{n(\mathrm{Cu})}}{1 + \dfrac{n(\mathrm{Cu_2O})}{n(\mathrm{Cu})}} \qquad \text{(S3)}$$

The calculated molar fractions of Cu_2O in the products obtained from various experimental conditions are shown in Table S4.

References for Supplementary Materials

1 Shapescribe. *Paste extruder - 10 cc v2.1 - extrudeuse a pâte*, (2014) Available at: http://www.thingiverse.com/thing:591849 (Accessed: 1st April 2016)

2 Wang, P. & Ngan, A. H. W. *Universal paste extruder 3ml syringe adaptor*, (2016) Available at: http://www.thingiverse.com/thing:1450092 (Accessed: 1st April 2016)

3 JCPDS - International Centre for Diffraction Data (2002)

4 Stadelmann, P. *JEMS-SAAS*, (2014) Available at: http://www.jems-saas.ch/ (Accessed: 1st April 2016)

5 Schneider, C. A., Rasband, W. S. & Eliceiri, K. W. NIH image to imagej: 25 years of image analysis. *Nat. Meth.* **9**, 671-675 (2012).
